# Brain Function Network: Higher Order vs. More Discrimination

**DOI:** 10.3389/fnins.2021.696639

**Published:** 2021-08-23

**Authors:** Tingting Guo, Yining Zhang, Yanfang Xue, Lishan Qiao, Dinggang Shen

**Affiliations:** ^1^School of Mathematics Science, Liaocheng University, Liaocheng, China; ^2^School of Biomedical Engineering, ShanghaiTech University, Shanghai, China; ^3^Shanghai United Imaging Intelligence Co., Ltd., Shanghai, China; ^4^Department of Artificial Intelligence, Korea University, Seoul, South Korea

**Keywords:** brain functional network, Pearson's correlation, mild cognitive impairment, gender prediction, higher-order correlation

## Abstract

Brain functional network (BFN) has become an increasingly important tool to explore individual differences and identify neurological/mental diseases. For estimating a “good” BFN (with more discriminative information for example), researchers have developed various methods, in which the most popular and simplest is Pearson's correlation (PC). Despite its empirical effectiveness, PC only encodes the low-order (second-order) statistics between brain regions. To model high-order statistics, researchers recently proposed to estimate BFN by conducting two sequential PCs (denoted as *PC*^2^ in this paper), and found that *PC*^2^-based BFN can provide additional information for group difference analysis. This inspires us to think about (1) what will happen if continuing the correlation operation to construct much higher-order BFN by *PC*^*n*^ (*n*>2), and (2) whether the higher-order correlation will result in stronger discriminative ability. To answer these questions, we use *PC*^*n*^-based BFNs to predict individual differences (Female vs. Male) as well as identify subjects with mild cognitive impairment (MCI) from healthy controls (HCs). Through experiments, we have the following findings: (1) with the increase of n, the discriminative ability of *PC*^*n*^-based BFNs tends to decrease; (2) fusing the *PC*^*n*^-based BFNs (*n*>1) with the *PC*^1^-based BFN can generally improve the sensitivity for MCI identification, but fail to help the classification accuracy. In addition, we empirically find that the sequence of BFN adjacency matrices estimated by *PC*^*n*^ (*n* = 1,2,3,⋯ ) will converge to a binary matrix with elements of ± 1.

## 1. Introduction

Brain functional network (BFN), learning from the resting-state functional magnetic resonance imaging (rs-fMRI), has become an increasingly important tool to understand the brain working mechanism (Liu et al., 2015; Jiang et al., 2020; Xue et al., 2020; Chen et al., 2021), reveal the biomarkers of neurological/mental disorders (Bijsterbosch et al., 2017; Li et al., 2017; Liu et al., 2017; Sun et al., 2020), and predict the individual differences (Dubois and Adolphs, 2016). However, establishing a “good” BFN (with more discriminative information for example) is currently a challenging problem, due to the low-quality of fMRI data and the high complexity of our brain.

In the past decades, numerous BFN construction methods (McLntosh and Gonzalez-Lima, 1994; Marrelec et al., 2006; Ramsey et al., 2010; Qiao et al., 2016; Li et al., 2017; Jiang et al., 2019) have been proposed. The main difference among these methods lies in the calculation of the relationship between different brain regions of interest (ROIs). Pearson's correlation (PC) is the commonly used method for constructing BFN due to its relative simplicity and reliability (Smith et al., 2013).

However, traditional PC cannot capture the complex relationship (e.g., non-linearity and non-Gaussianity) among ROIs because it can only encode the simple linear correlation (Wan et al., 2006; Xie et al., 2008; Zhang et al., 2016). In contrast, the two sequential PCs (denoted as *PC*^2^ in this paper) provide a high-order statistic that, in theory, can encode more complex relationship (Swami et al., 1997; Zhou et al., 2018). In fact, *PC*^2^ has been used to estimate BFN and some findings have been obtained in recent years (Chen et al., 2016; Zhang et al., 2016; Zhou et al., 2018). For instance, Chen et al. (2016) revealed that *PC*^2^ can encode the complex interactions between different ROIs, and Zhang et al. (2016) found that *PC*^2^ is more sensitive to AD identification.

**Figure 1:**
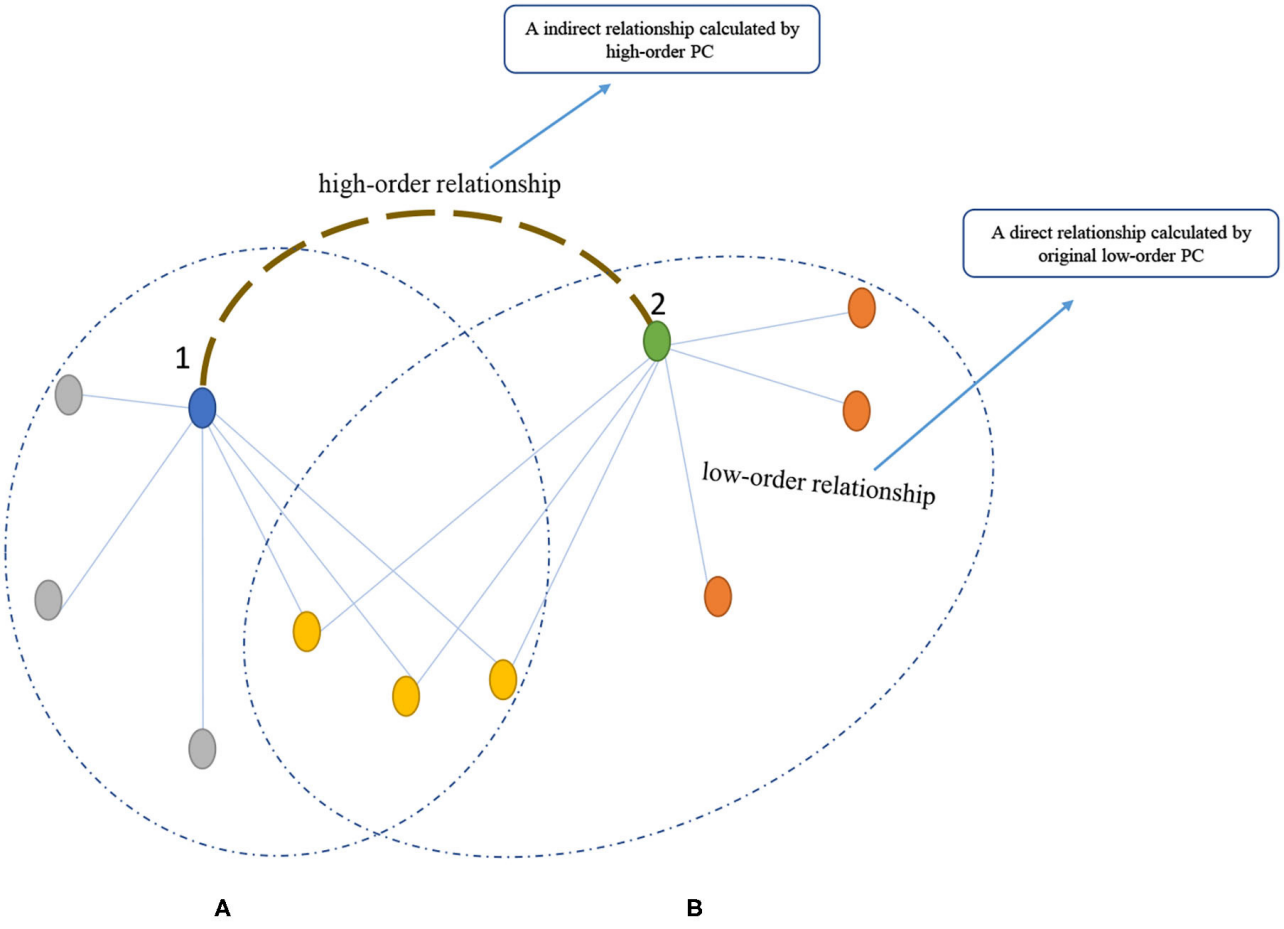
An analogy of *PC*^2^ to a friendship network. The **(A,B)** are the friendship networks of nodes 1 and 2, respectively.

In order to have an intuitive explanation of the original low-order PC and high-order PC (i.e., *PC*^2^), we provide a simple analogy via a friendship network in Figure 1, where each node corresponds to a person and each edge means existence of friendship between two nodes. As shown in Figure 1, we note that there is no direct edge between Node 1 and Node 2, meaning that these two persons have no relationship in a low-order form. However, from a high-order view, Nodes 1 and 2 may have some kinds of relationship since they share three common friends. Such a friend-sharing relationship (with similar friend circles) can be easily captured by the high-order PC, according to its definition (i.e., two sequential correlations). This indicates that the high-order PC can find more complex relationship that cannot be directly modeled in a low-order way.

Inspired by the potential powerfulness of *PC*^2^, a natural question is (1) what will happen if continuing the correlation operation to construct much higher-order BFN by *PC*^*n*^ (*n*>2), and (2) whether the higher-order correlation will result in stronger discriminative ability or other useful information.

For investigating the above problems, we construct *PC*^*n*^-based BFNs on two public datasets, and then apply the constructed BFNs to conduct the classification task. Through the experiments, we get two interesting findings: (1) for the *PC*^*n*^-based BFNs, with the increase of *n*, their discriminative ability tends to decrease; (2) by fusing the *PC*^*n*^-based BFNs (*n*>1) with the original *PC*-based BFN, the sensitivity for MCI identification is generally increased, but the classification accuracy has no significant improvement. In addition, we have empirically found that the BFN adjacency matrix sequence constructed by *PC*^*n*^ (*n* = 1,2,3 ⋯ ) is convergent, where *PC*^1^corresponds to the original PC.

The rest of this paper is organized as follows. In section 2, we introduce the data acquisition, data preprocessing and the *PC*^*n*^-based BFN construction scheme. In section 3, we design two classification tasks for evaluating the proposed methods. In section 4, we discuss our findings and analyze the convergence of the *PC*^*n*^-matrix sequence, and also introduce several limitations of this work with possible research plans in the future. Finally, we briefly summarize this paper in section 5.

## 2. Materials and Methods

### 2.1. Data Acquisition

Two public datasets, from Alzheimer's Disease Neuroimaging Initiative (ADNI)^1^ and Human Connectome Project (HCP)^2^, respectively, are used in our experiments. For ADNI dataset, we first remove the subjects if (1) the maximum translation/rotation of the head motion exceeds 2.0, or (2) the volumes with big frame-to-frame displacement (>0.5) accumulate more than 2.5 min (Power et al., 2012), which leaves us 299 scans (including 154 HCs and 145 MCIs) from 143 participants (with some participants scanned at one or more times). The scanning parameters are given as follows: the image resolution is from 2.29 to 3.31 mm for in-plane and the slice thickness equal to 3.31 mm, TE (echo time) = 30 ms, TR (repetition time) is from 2.2 to 3.1 s, flip angle = 80^0^, field of view (FOV) = 198.75 × 212 mm^2^, matrix size = 64 × 64, voxel size = 3.3 × 3.3 × 3.3mm^3^ and the scanning time for each subject is 7 min (resulting in 140 volumes) (Wang et al., 2019). The demographic information of 143 subjects is shown in Table 1.

**Table 1:** The main demographic information of 143 subjects.

**Category**	**Scan #**	**Age (Years)**	**Gender (M/F)**
MCI	145	71.99 ± 7.67	95/50
HC	154	75.36 ± 6.16	67/87

For HCP dataset, 1,003 subjects, including 469 males and 534 females, are involved in our experiment. Each subject was scanned in four sessions, each of which generates 1,200 volumes, thus resulting in 4,800 volumes. Data acquisition is performed as follows: TR = 720 ms, TE = 33.1 ms with 72 slices, flip angle = 52^0^, FOV = 208 × 180 mm^2^, matrix size = 104 × 90, and voxel size = 2 × 2 × 2 mm^3^ (WU-Minn, 2017). The demographic information of 1,003 subjects is shown in Table 2.

**Table 2:** The main demographic information of 1, 003 subjects.

**Category**	**Scan #**	**Age (Years)**
Female	534	22–36+
Male	469	22–36+

### 2.2. Data Preprocessing

For each subject from ADNI dataset, we discard the first three volumes for magnetization balance, and then use the FSL FEAT software^3^ for preprocessing the remaining 137 volumes, including slice timing correction, head motion estimation, bandpass filtering, and regression of nuisance covariates (white matter, cerebrospinal fluid, and motion parameters). After that, we align the fMRIs (that are skull-stripped based on T1-weighted MRI) onto the Montreal Institute of Neurology (MNI) space, and perform spatial smoothing by the Gaussian kernel with full-width-at-half-maximum (FWHM) of 6 mm. Finally, we divide the preprocessed volumes into 116 ROIs based on the automatic anatomical labeling (AAL) template (Tzourio-Mazoyer et al., 2002), and extract its mean blood oxygen level dependent (BOLD) signals (fMRI series) (Wang et al., 2019).

For the subjects from HCP dataset, they are pre-processed according to WU-Minn (2017). More specifically, structural noise in the fMRI was removed through the pairsed independent component analysis (ICA) (Beckmann and Smith, 2004) with FMRIB's ICA-based Xnoiseifier (FIX) in FSL toolbox (Griffanti et al., 2014). Then, inter-subject registration of cerebral cortex was carried out using areal-feature-based alignment and Multimodal Surface Matching algorithm (MSMAII) (Smith et al., 2011; Glasser et al., 2016). In order to divide the ROIs of the brain, the data was first temporally normalized and fed into group-PCA (Beckmann and Smith, 2004; Smith et al., 2014), and then the group-ICA based on FSL's MELODIC tool (Griffanti et al., 2014) was applied to the data obtained by group-PCA (Beckmann and Smith, 2004). Note that the data dimension after group ICA determines the number of ROIs, which is simply set to 100 in this paper.

### 2.3. *PC*-Based BFN Estimation

As described earlier, PC is the simplest and most popular approach to construct BFNs (Smith et al., 2013). The edge weight between the ith and jth ROIs of *PC*-based BFN is defined as follows:

(1) $$\begin{array}{r} r_{ij}=\frac{{\left(x_{i}-{\bar{x}}_{i}\right)}^{T}\left(x_{j}-{\bar{x}}_{j}\right)}{\sqrt{{\left(x_{i}-{\bar{x}}_{i}\right)}^{T}\left(x_{i}-{\bar{x}}_{i}\right)}\sqrt{{\left(x_{j}-{\bar{x}}_{j}\right)}^{T}\left(x_{j}-{\bar{x}}_{j}\right)}} \end{array}$$

where $x_{i}\in R^{M}$is the fMRI signal from the ith ROI, and ${\bar{x}}_{i}\in R^{M}$ is a constant signal obtained by averaging the elements in *x*_*i*_. Without loss of generality, we suppose that *x*_*i*_ is centralized by $x_{i}-{\bar{x}}_{i}$ and normalized by $\sqrt{{\left(x_{i}-{\bar{x}}_{i}\right)}^{T}\left(x_{i}-{\bar{x}}_{i}\right)}$. Then, Equation ( 1) can be simplified to $r_{ij}={\left(x_{i}\right)}^{T}x_{j}$, and its matrix form is defined as follows:

(2) $$\begin{array}{r} R=X^{T}X \end{array}$$

where $X=\left[x_{1},x_{2},\cdots \;,x_{N}\right]\in R^{\left(M\times N\right)}$ is the data matrix, and *R* is the edge weight matrix of *PC*-based BFN. For the convenience of presentation in what follows, we simply call the original PC as *PC*^1^, and the corresponding edge weight matrix of *PC*-based BFN as *R*_1_ = *R*.

### 2.4. From *PC*^1^ to *PC*^*n*^

**Figure 2:**
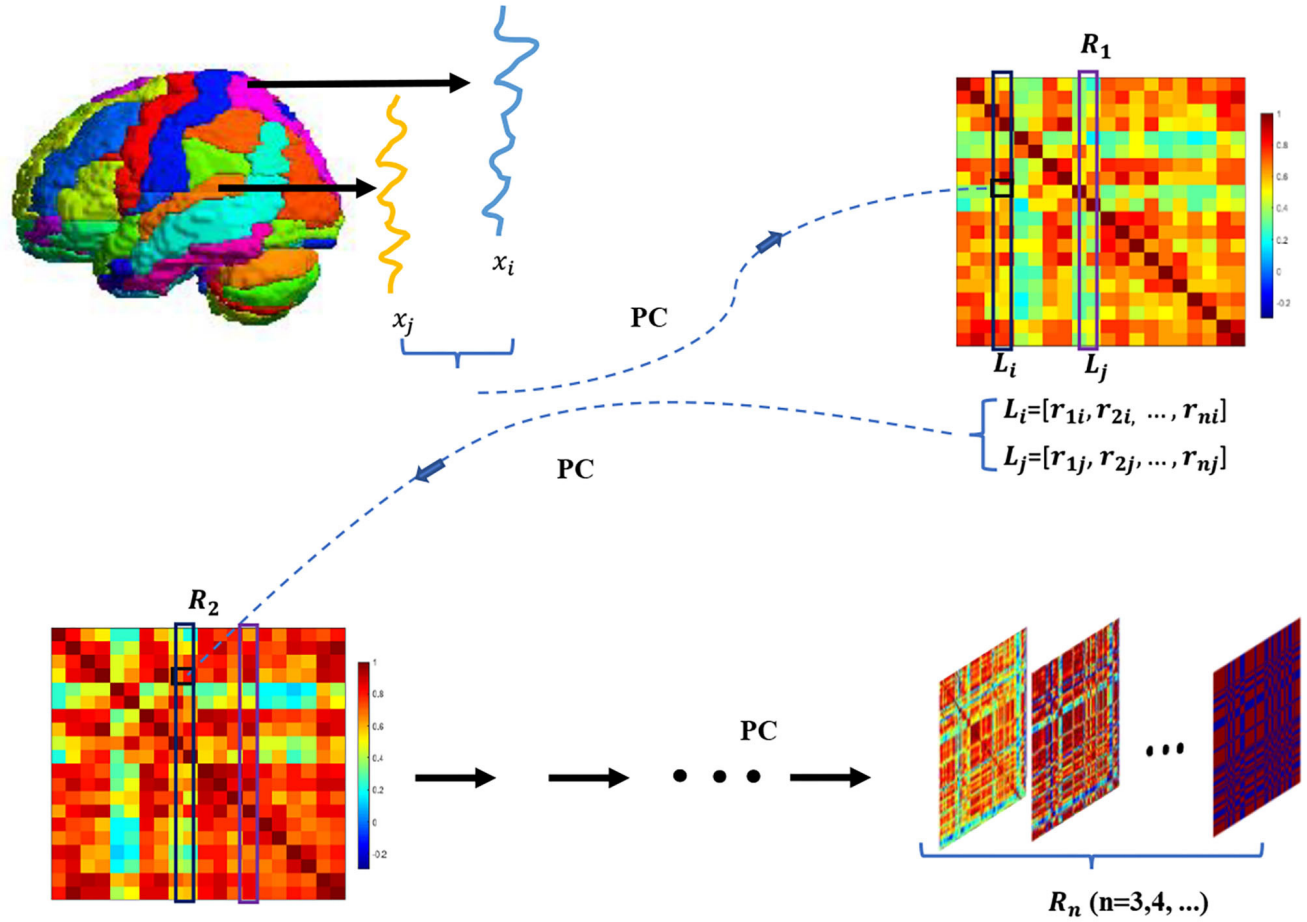
The pipeline of building *R*_*n*_. *R*_1_ is first obtained via computing PC coefficient of the BOLD signals associated with different ROIs. Then, *R*_1_ is seen as the new data matrix to calculate *R*_2_, where the feature vector *L*_*i*_ is considered as a new “signal” that indicates the correlation between the ith ROI and other ROIs. By continuously conducting the above operation, we calculate *R*_*n*_ (*n* = 2,3,⋯ ).

Despite its simplicity and popularity, *PC*^1^-BFN can only model the low-order statistics that may be insufficient to capture more complicated relationship between ROIs. As an alternative to *PC*^1^, Zhang et al. (2016) recently proposed to construct the high-order BFN by correlation's correlation. Specifically, as shown in Figure 2, the *PC*^1^-based BFN (i.e., *R*_1_) is first established by Equations ( 1) or ( 2). Then, the *R*_1_ is used as a data matrix for constructing the high-order *PC*^2^-based BFN (i.e., *R*_2_) by considering each row (or column) of *R*_1_ as a new “signal” associated with each ROI. Interestingly, the researchers find that *R*_2_ can provide additional information in group difference analysis (Zhang et al., 2016).

Motived by *PC*^2^, in this paper we go further to construct higher-order *PC*^*n*^-based BFN (i.e., *R*_*n*_, *n*>2) by continuously conducting the correlation operation. As a result, we obtain a sequence of BFN adjacency matrices, and this sequence from *R*_1_ to *R*_*n*_ can be achieved by the following iteration formula (please see Appendix for the details of formula derivation):

(3) $$\begin{array}{r} R_{n}=\frac{R_{n-1}CR_{n-1}}{\sqrt{diag\left(R_{n-1}CR_{n-1}\right)}\sqrt{diag\left(R_{n-1}CR_{n-1}\right)}},n=2,3,4,\cdots \end{array}$$

where $C=I-\frac{E}{n}$ is a constant matrix (with *I* and *E* being the identity and all-one matrix, respectively), and *diag*(*A*) is a diagonal matrix that shares the same diagonal elements with *A*.

## 3. Experiment

In this section, we conduct experiments to answer the questions about the higher-order BFNs: (1) whether the higher-order BFN are more discriminative for achieving better classification performance, and (2) by combinating with *PC*^1^-based BFN, whether the *PC*^*n*^-BFNs (*n*>2) can provide additional information as *PC*^2^-based BFNs.

### 3.1. Experimental Setting

**Figure 3:**
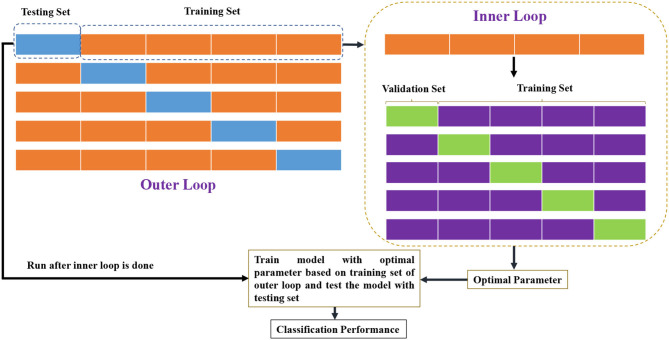
The pipeline of 5-F CV in this study.

In this experiment, the classification performance is evaluated by five-fold subject-level cross validation (5-F CV), to ensure that fMRI scans of the subject do not appear in both training and testing sets (Wong and Tzu-Tsung, 2015). Specifically, as shown in Figure 3, the 143 (or 1,003) subjects of ADNI (or HCP) are first divided into five-fold, each of which contains almost the same number of subjects. Then, four-fold are used to train the classifier, and the remaining one is used to test the classification performance. Linear support vector machine (SVM) (Chang and Lin, 2011) with default parameter C = 1 is utilized to perform the classification task. The edge weights of the BFN are adopted as features for classification. In consideration of the fact that the number of edges far exceeds the sample size, *t*-test is used to select edge weight features prior to the classification task. Two binary classification tasks are conducted to evaluate the constructed BFNs with different orders. One is to identify subjects with MCI from HCs based on the ADNI dataset, and the other is to predict gender based on the HCP dataset.

**Figure 4:**
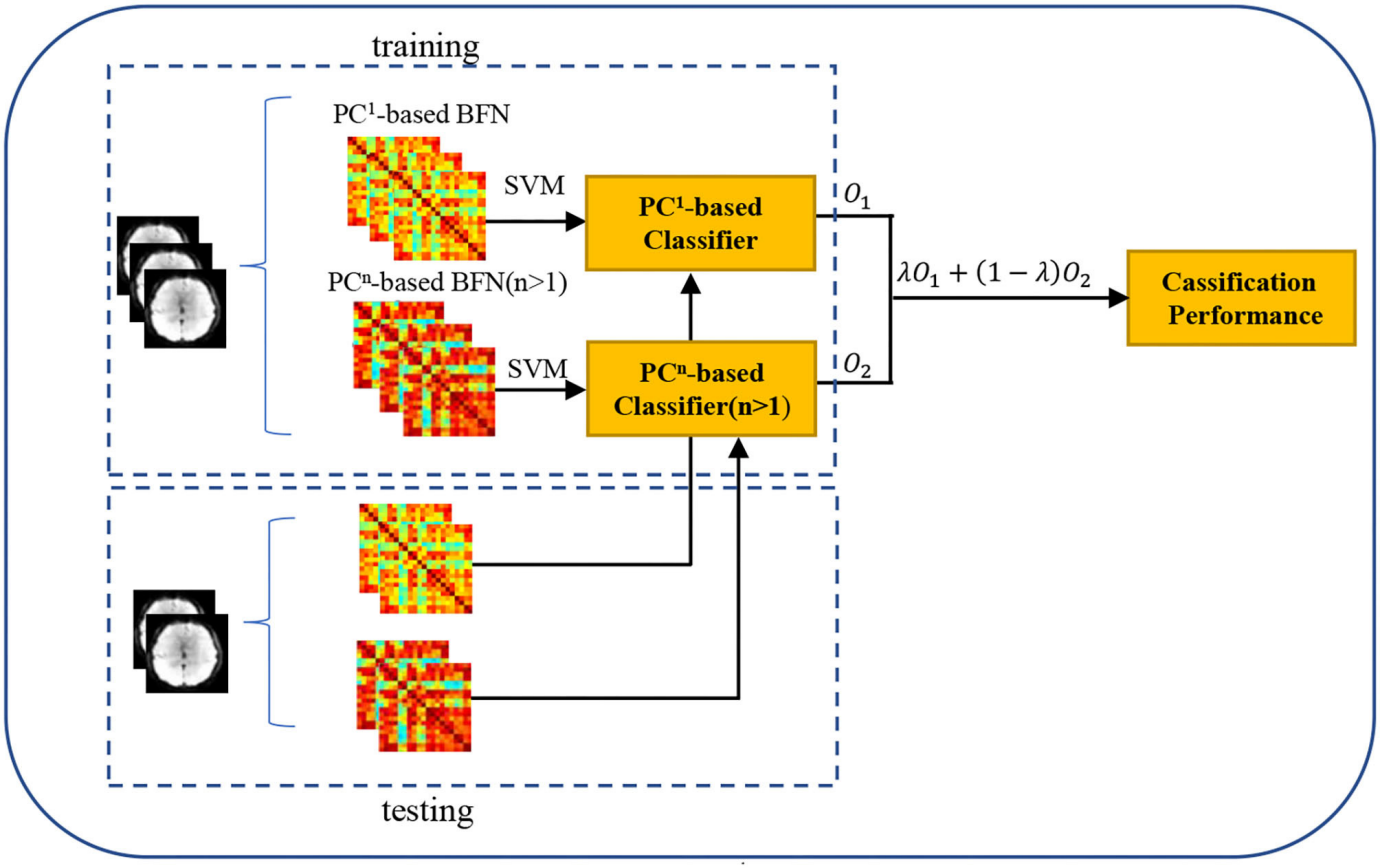
The classification pipeline based on the *PC*^1^- and *PC*^*n*^ (n>1)-based BFNs. We first train two classifiers based on the *PC*^1^- and *PC*^*n*^ (*n*>1)-based BFNs, respectively, and then fuse the outputs (i.e., *O*_1_ and *O*_2_) of the two classifiers via λ*O*_1_+(1−λ)*O*_2_, where λ is the optimal parameter obtained by an inner 5-F CV.

Furthermore, we design experiment to verify whether the *PC*^*n*^-BFNs (*n*>2) can provide additional information by combining the *PC*^*n*^- and *PC*^1^-based BFNs. In particular, we first train two classifiers (SVM with C = 1) based on *PC*^1^- and *PC*^*n*^ (*n*>1)-based BFNs, respectively, and then fuse the outputs of these two classifiers. As shown in Figure 4, the outputs, *O*_1_ and *O*_2_, of the two classifiers measure the probabilities that the current subject belongs to the positive class. With the two probabilistic outputs, we can achieve the fused classification result via λ*O*_1_+(1−λ)*O*_2_, where the optimal parameter λ is selected in the range of [0.1, 0.2, 0.3, 0.4, ⋯ , 0.9] by an inner 5-F CV on the training set. Finally, we retrain the classifier with the optimally selected parameter, and put the BFNs of the testing subject into the trained classifiers for the ultimate performance as shown in Figure 3.

### 3.2. Estimated Functional Brain Networks

In the first group of experiments, we visualize the sequence of BFN constructed by *PC*^*n*^.

**Figure 5:**
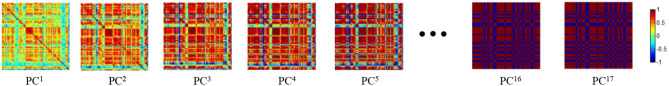
The visualization of BFNs with different order *n*. We empirically note that the adjacency matrix sequence will converge after dozens of iterations.

In Figure 5, we randomly select a subject from ADNI dataset and visualize the BFNs constructed by different methods. It can be observed that the BFNs estimated by *PC*^16^ and *PC*^17^ are almost the same, indicating that the adjacency matrix sequence may converge to a binary matrix with entries of 1 and −1. This phenomenon motivates us to explore and prove it in theory. In the discussion section, we will further analyze and discuss this problem.

### 3.3. Experiment Results

In this group of experiments, two binary classification tasks (i.e., MCI vs. HC, Female vs. Male) based on the constructed BFNs with different orders are first conducted to evaluate whether the higher-order BFN are more discriminative, and then to verify whether the *PC*^*n*^-BFNs (n>2) can provide additional information by combining the *PC*^*n*^- and *PC*^1^-based BFNs. We evaluate the classification performance of different methods by a set of quantitative measures, including accuracy (ACC), sensitivity (SEN), and specificity (SPE). Their expressions are defined as follows:

(4) $$\begin{array}{r} ACC=\frac{TP+TN}{TP+TN+FN+FP} \end{array}$$

(5) $$\begin{array}{r} SEN=\frac{TP}{TP+FN} \end{array}$$

(6) $$\begin{array}{r} SPE=\frac{TN}{TN+FP} \end{array}$$

where TP, TN, FP, and FN denote the number of true positive, true negative, false positive, and false negative numbers, respectively.

**Figure 6:**
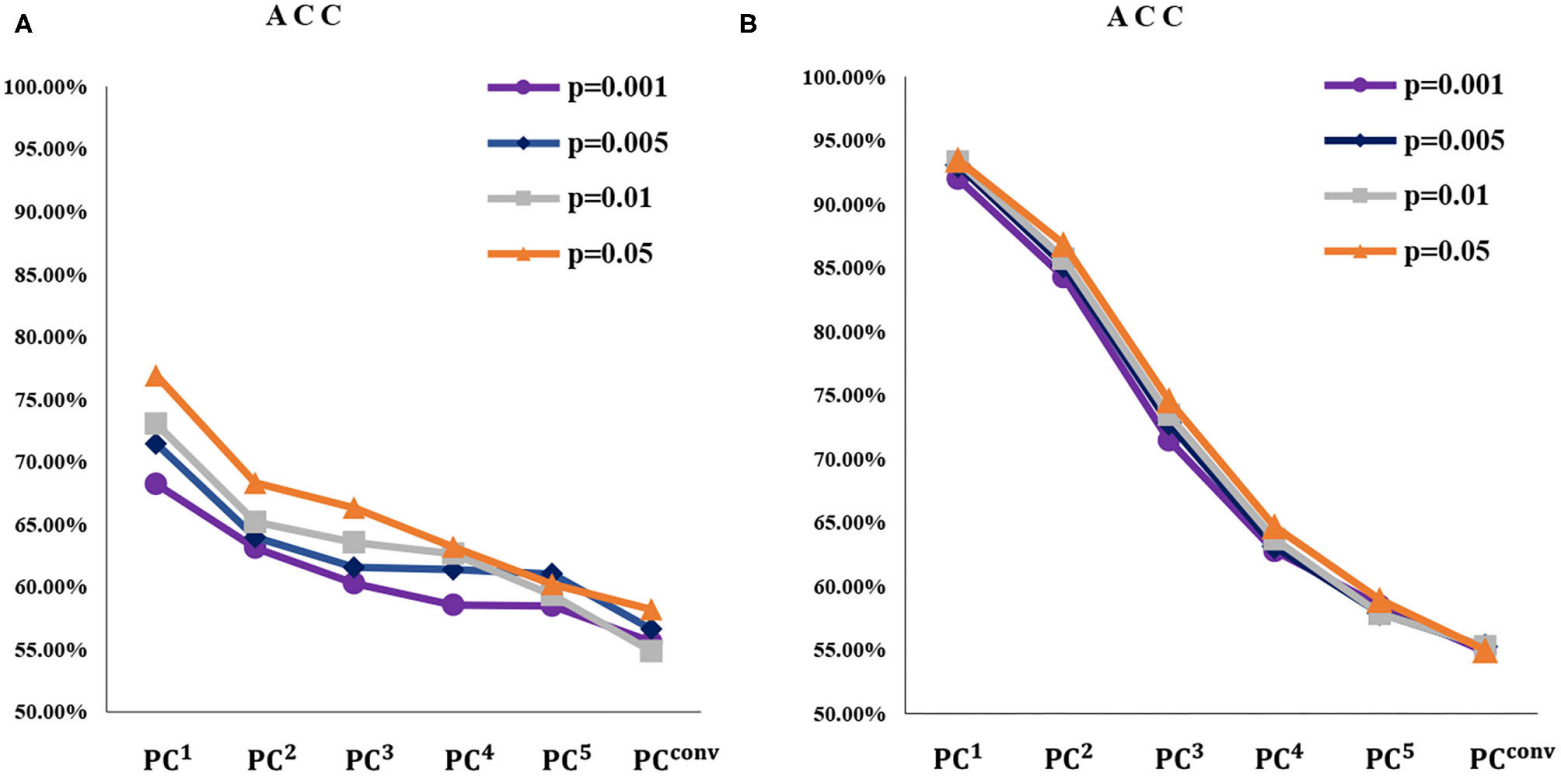
The ACC of **(A)** MCI identification and **(B)** gender prediction based on BFNs with six different orders and four different *p*-values (for filtering features). The *PC*^*conv*^-based BFN is the empirically convergent adjacency matrix through thirty iterations. Note that, with the increase of *n*, the ACC of *PC*^*n*^-based BFN tends to decrease in all the cases.

**Figure 7:**
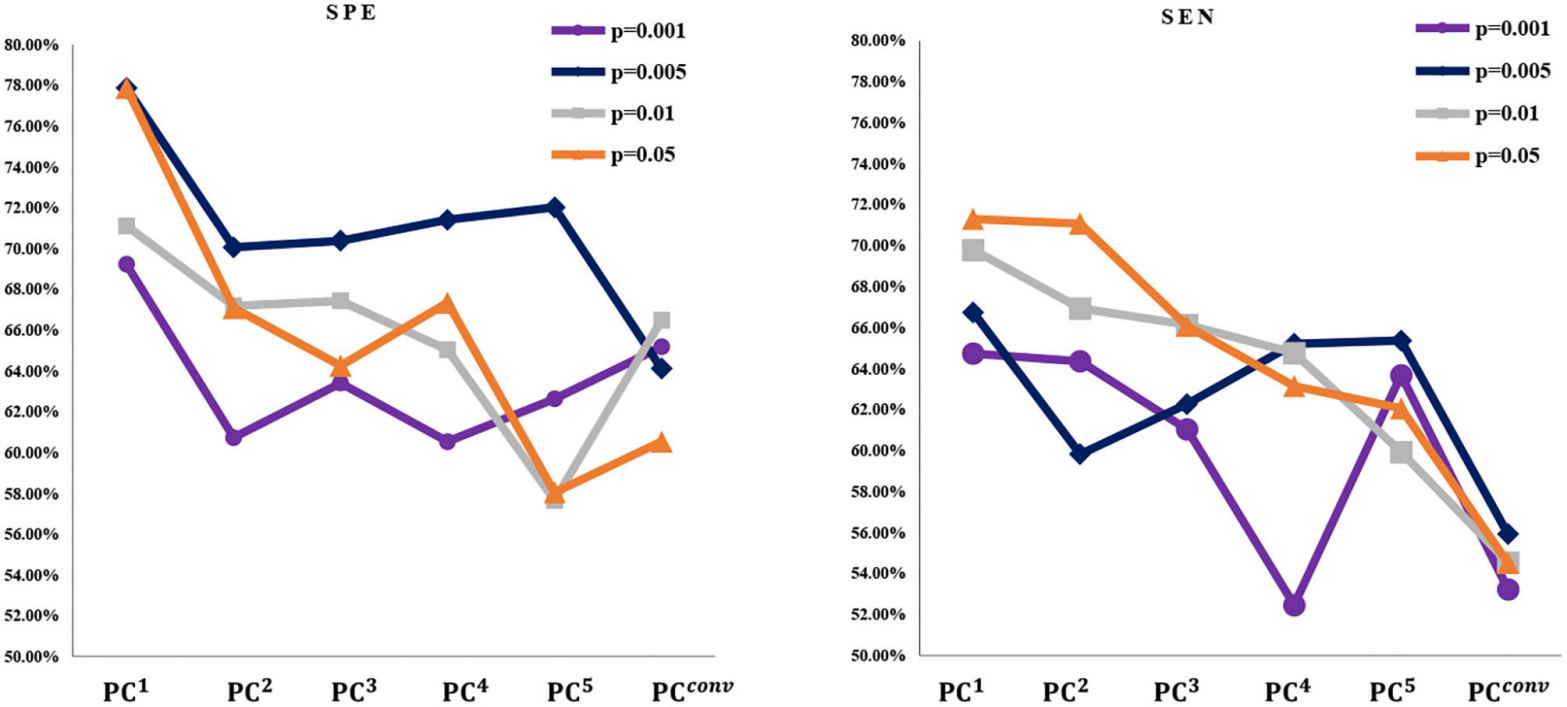
The SEN and SPE based on six kinds of BFNs for MCI identification, respectively. It can be observed that the SEN and SPE of *PC*^1^ is better than other cases. In addition, under different *p*-values of *t*-test, the SEN and SPE tend to change greatly.

As described in the experimental setting, we construct BFNs with different orders via multiple sequential PCs and apply them in the classification task. The classification results on two datasets are reported in Figures 6, 7.

**Figure 8:**
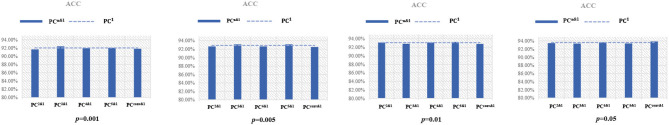
The ACC of gender prediction based on the fusion of *PC*^*n*^-based BFN and *PC*^1^-based BFN under different *p*-values. The *PC*^*n&*1^ corresponds to the fused classification accuracy of *PC*^*n*^ and *PC*^1^, *n* = 2,3,4, convergence(conv). The dotted line is the ACC of *PC*^1^ itself (without fusion with *PC*^*n*^).

**Figure 9:**
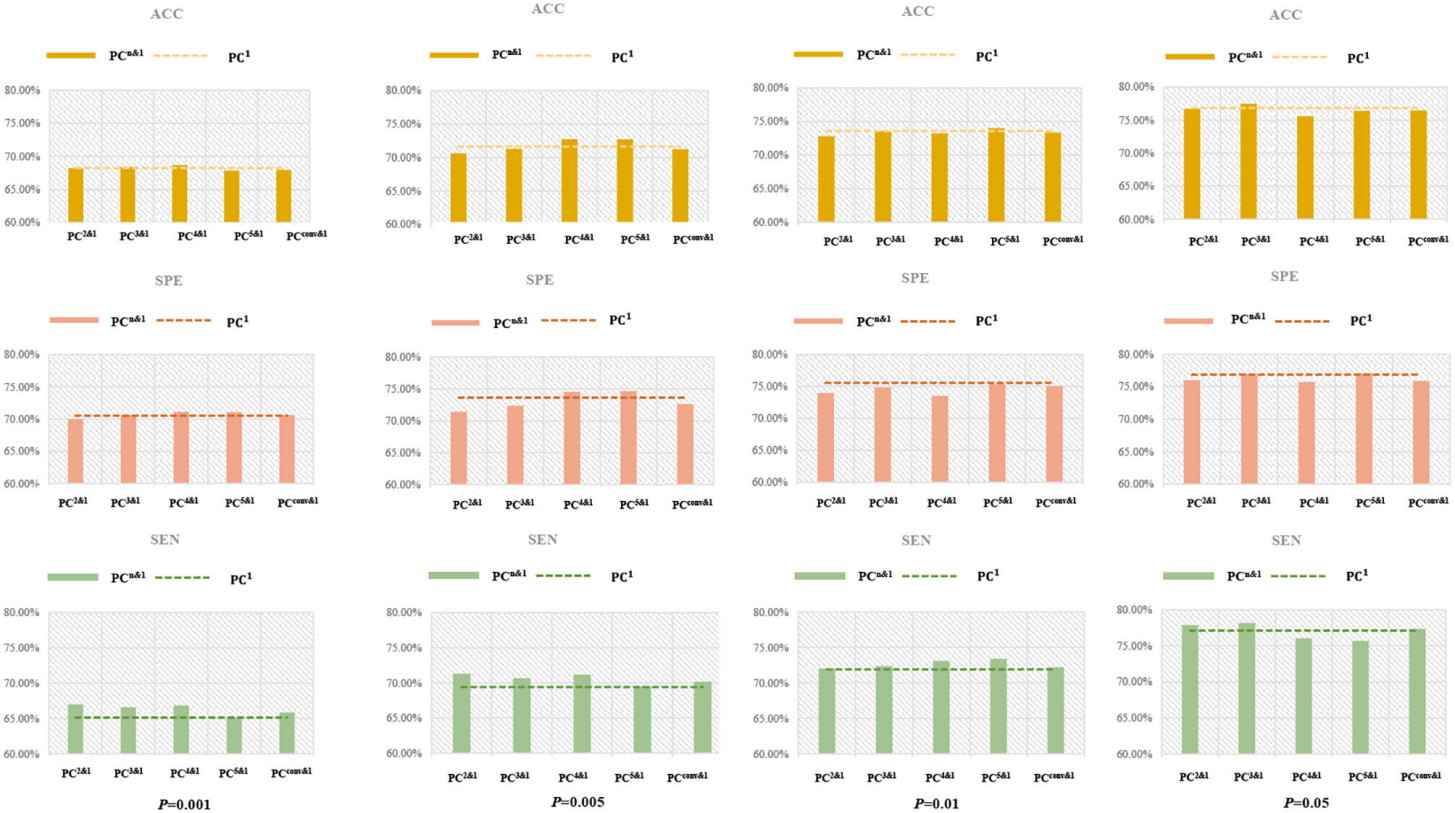
The performance (ACC, SPE, and SEN) for MCI identification based on fusion of *PC*^*n*^-based BFN and *PC*^1^-based BFN under different *p*-values. The *PC*^*n&*1^ corresponds to the fused performance of *PC*^*n*^ and *PC*^1^, *n* = 2,3,4, conv. The dotted line is the performance of *PC*^1^ itself (without fusion with *PC*^*n*^). We can observe that the fusion of low- and higher-order BFNs fails to improve the ACC and SPE in most cases, but generally improves SEN.

Furthermore, the classification results based on the fusion of the low-order and higher-order BFNs are reported in Figures 8, 9, respectively.

## 4. Discussion

In this section, we first study the discrimination of features and the additional information provided by higher-order BFN. Then, we further analyze the convergence of BFNs with different orders and show some related findings about it. Finally, we illustrate the limitations of the current work and the direction of future work.

### 4.1. Discrimination of Higher-Order BFN

Based on the classification results, we analyze the discrimination of higher-order BFN. Based on Figures 6, 7.

(1) As *n* increases, the ACC of *PC*^*n*^ generally decreases for both MCI identification and gender prediction, even with various *p*-values in *t*-test for selecting different groups of feature. This illustrates that the higher-order does not result in stronger discriminative ability. Especially when the BFN converges with a big n, the ACC approaches 50%, meaning that its discrimination almost disappears since our task is a binary classification.

(2) Different from the ACC that decreases monotonically, the SPE and SEN generally fluctuate for the MCI identification task under most *p*-values. However, the overall performance shows a downtrend for both SPE and SEN. This further illustrates that the higher-order BFNs in the sense of multiple correlations do not necessarily help the discrimination.

(3) As a byproduct of our experiments, we find that the *p*-value generally has a great impact on the final performance. For example, as shown in Figure 6, the *PC*^*n*^-based BFNs have better performance when the *p*-value is 0.05. In terms of the fused classification results, it can also be observed that *p* = 0.05 will result in the best ACC, SEN, and SPE, as shown in Figure 9. This suggests that we should carefully select features (edge weights) prior to the classification.

**Figure 10:**
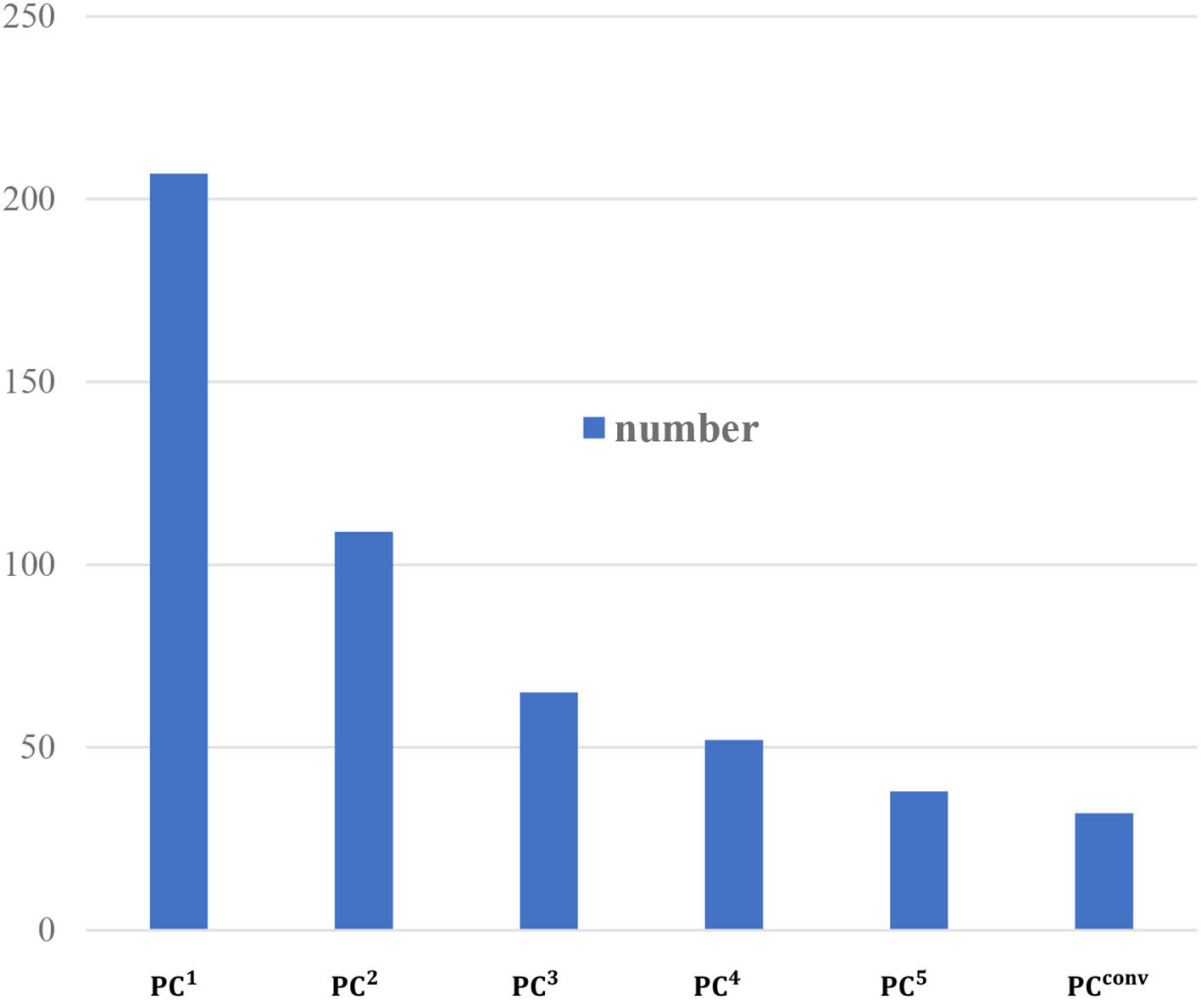
The number of selected features with *p*-values = 0.001.

In order to better explain our classification results, we further investigate the number of features involved in the *PC*^*n*^-based MCI classification, and report the results with *p*-value of 0.001 in Figure 10.

It can be seen, from Figure 10, that *PC*^1^ includes the largest number of features (207 features) and the number of features selected by *PC*^*n*^ decrease with the increase of *n*. This means that the higher-order BFN instead contains less discriminative information than the low-order BFN.

### 4.2. Supplementary Information of Higher-Order BFNs

The higher-order BFNs can provide some additional information for the classification task. As shown in Figure 9, although it cannot benefit the ACC and SPE, the combination of *PC*^*n*^-based BFN and *PC*^1^-based BFN can generally improve the SEN. The improvement of SEN is generally important, because it may help to detect the brain disorders in their early stage. In addition, this also give a reason to believe that *PC*^*n*^(*n*>1) would provide potentially additional information for encoding complex relationship between different ROIs.

### 4.3. Convergence Analysis

In the process of constructing the BFN based on the sequential PCs, we have an interesting finding that the sequence of BFN adjacency matrices estimated by *PC*^*n*^ (*n* = 1,2,3⋯ ) will converge to a binary matrix with entries of 1 and −1 as shown in Figure 5. Although we cannot provide a direct explanation of this phenomenon from a biological mechanism, it will inspire us to think whether the interaction between different ROIs promotes or inhibits each other at a certain working stage of the brain.

Currently, the convergence of the BFN sequence cannot be rigorously proved in theory. We will explore this problem in the future work. However, in the process of searching the rigorous proof of convergence, we achieve several interesting theoretical findings. In particular, let ${\left\{R_{n}\right\}}_{n=1}^{\infty }$ be the sequence of correlation coefficient matrices generated by the iterative formula shown in Equation ( 3), and then we have the following results or conjecture:

1. After iterating the matrix for a certain number of times, the elements in the matrix will be monotonic with the number of iterations, and the sign of a small number of elements will change once.
2. Supposing that the rank of *R*_*n*_ is $r_{n}^{*}$, we can achieve a sequence of rank ${\left\{r_{n}^{*}\right\}}_{n=1}^{\infty }$ that is monotonically decreasing, since the rank of the matrix product is not greater than the rank of any one of them (Lancaster and Tismenetsky, 1985).
3. According to Schauder's fixed-point theorem (Kellogg, 1976), we find that the iterative formula given in Equation ( 3) has a fixed-point^4^.

### 4.4. Limitations and Future Work

First, in this paper we only study the HOBFN constructed by multiple sequential PCs. In fact, however, many traditional BFN estimation methods such as sparse representation (Lee et al., 2011) can be extended to higher-order versions through the similar way (i.e., multiple sequential calculations). In the future, we plan to discuss more kinds of HOBFNs and systematically compare their discrimination. Second, we cannot rigorously prove the convergence of matrix sequence in theory, and the biological mechanism of HOBFN is unclear at present. We will try to prove its convergence and explore its biological mechanism in the future.

## 5. Conclusion

Recent researches have proposed many methods to construct high-order BFNs (HOBFNs) that can generally provide some additional information for poring cerebral mechanisms. For instance, Guo et al. (2017) constructed HOBFN using minimum spanning tree and Zhang et al. (2016) recently proposed to construct BFNs by conducting two sequential PCs. In this paper, we mainly focus on constructing the *PC*^*n*^-based BFN by multiple sequential PCs, and exploring its performance in the classification tasks. Through experiments, we find that the higher-order does not necessarily result in stronger discriminative ability, but the additional information provided by *PC*^*n*^ (n>1) is helpful for *PC*^1^ to improve the sensitivity of the classifier. In addition, we empirically find that the matrix sequence constructed by *PC*^*n*^ (n=1,2,3 ⋯ ) converges to a matrix with elements of −1 and 1.

## Data Availability Statement

The original contributions generated for the study are included in the article/supplementary material, further inquiries can be directed to the corresponding author/s.

## Ethics Statement

The studies involving human participants were reviewed and approved by public datasets HCP and ADNI. The patients/participants provided their written informed consent to participate in this study.

## Author Contributions

LQ and TG contributed to the conception and design of this research. LQ, YZ, and TG derived and designed the theoretical and experimental parts in the article. TG, LQ, YX, and DS wrote the first draft of the manuscript. All authors participated in the revision, reading, and approval of the manuscript.

## Conflict of Interest

DS was employed by the company Shanghai United Imaging Intelligence Co., Ltd. The remaining authors declare that the research was conducted in the absence of any commercial or financial relationships that could be construed as a potential conflict of interest.

## Publisher's Note

All claims expressed in this article are solely those of the authors and do not necessarily represent those of their affiliated organizations, or those of the publisher, the editors and the reviewers. Any product that may be evaluated in this article, or claim that may be made by its manufacturer, is not guaranteed or endorsed by the publisher.

## Footnotes

^1^http://adni.loni.ucla.edu.

^2^http://www.humanconnectomeproject.org.

^3^http://www.fmrib.ox.ac.uk/fsl.

^4^Schauder fixed point: suppose X is a banach space, and *M*∈*X* is a bounded closed convex set. If F: *M*→*M* is a compact-operator, there is *x*∈*M* such that F(x)=x.

## Appendix

### Construction Details of the Iterative Formula

Suppose that $R_{1}=\left(r_{ij}\right)\in R^{\left(n\times n\right)}$ is an initial Pearson's correlation coefficient matrix estimated from observed data of an n-dimensional random vector *X* = (*X*_1_, *X*_2_, ⋯ , *X*_*n*_). If considering *R*_1_ as new observed data, each row (or equivalently each column, due to the symmetry) of which corresponds to a random variable in *X*, we can estimate another correlation coefficient matrix *R*_2_ based on *R*_1_. Further, by continuously conducting the operation of “correlation's correlation,” we can have a sequence of correlation coefficient matrices ${\left\{R_{n}\right\}}_{n=1}^{\infty }$, and we empirically find that the matrix sequence will converge, if all the involved calculations are legal (for example, the denominator is not zero).

In order to prove the conjecture of convergence, we first express the sequence of correlation's correlation as an iterative formula. Specifically, let *r*_*i*_ and *r*_*j*_ be the *ith* and *jth* rows of current correlation matrix $R=\left(r_{ij}\right)\in R^{\left(n\times n\right)}$, respectively. Then, the Pearson's correlation coefficient, denoted by $r_{ij}^{new}$, between the corresponding variables is given as follows,

(7) $$r_{ij}^{new}=\frac{(r_{i}-\bar{r_{i}1}){\left(r_{j}-\bar{r_{j}1}\right)}^{T}}{∥(r_{i}-\bar{r_{i}1}∥∥r_{j}-\bar{r_{j}1}∥}$$

where ${\bar{r}}_{i}=\frac{\Sigma_{k=1}^{n}r_{ik}}{n}$, ${\bar{r}}_{j}=\frac{\Sigma_{k=1}^{n}r_{jk}}{n}$ (*i, j* = 1, 2, ⋯ , *n*), and 1∈*R*^(1 × *n*)^ is an all-one vector. As a result, the new correlation matrix $R^{new}=r_{ij}^{new}$ can be expressed as follows:

(8) $$\begin{array}{r} R^{new}=\left[\begin{array}{c} \frac{r_{1}-{\bar{r}}_{1}1}{∥r_{1}-{\bar{r}}_{1}1∥}\\ \vdots \\ \frac{r_{n}-{\bar{r}}_{n}1}{∥r_{n}-{\bar{r}}_{n}1∥}\\ \; \end{array}\right]\left[\begin{array}{c} {\left(\frac{r_{1}-{\bar{r}}_{1}1}{∥r_{1}-{\bar{r}}_{1}1∥}\right)}^{T},\cdots \;,{\left(\frac{r_{n}-{\bar{r}}_{n}1}{∥r_{n}-{\bar{r}}_{n}1∥}\right)}^{T} \end{array}\right]\\ =\left[\begin{array}{ccc} \frac{1}{∥r_{1}-{\bar{r}}_{1}1∥} & \cdots & 0\\ \vdots & \ddots & \vdots \\ 0 & \cdots & \frac{1}{∥r_{n}-{\bar{r}}_{n}1∥} \end{array}\right]\left[\begin{array}{c} r_{1}-{\bar{r}}_{1}1\\ \vdots \\ r_{n}-{\bar{r}}_{n}1 \end{array}\right]\\ \left[\begin{array}{c} {\left(r_{1}-{\bar{r}}_{1}1\right)}^{T},\cdots \;,{\left(r_{n}-{\bar{r}}_{n}1\right)}^{T} \end{array}\right]\left[\begin{array}{ccc} \frac{1}{∥r_{1}-{\bar{r}}_{1}1∥} & \cdots & 0\\ \vdots & \ddots & \vdots \\ 0 & \cdots & \frac{1}{∥r_{n}-{\bar{r}}_{n}1∥} \end{array}\right] \end{array}$$

Note that

(9) $$\begin{array}{r} \left[\begin{array}{ccc} \frac{1}{∥r_{1}-{\bar{r}}_{1}1∥} & \cdots & 0\\ \vdots & \ddots & \vdots \\ 0 & \cdots & \frac{1}{∥r_{n}-{\bar{r}}_{n}1∥} \end{array}\right]\\ =\frac{1}{\sqrt{diag\left(\left[\begin{array}{c} r_{1}-{\bar{r}}_{1}1\\ \vdots \\ r_{n}-{\bar{r}}_{n}1 \end{array}\right]\left[\begin{array}{c} {\left(r_{1}-{\bar{r}}_{1}1\right)}^{T},\cdots \;,{\left(r_{n}-{\bar{r}}_{n}1\right)}^{T} \end{array}\right]\right)}} \end{array}$$

(10) $$\begin{array}{r} \left[\begin{array}{c} r_{1}-{\bar{r}}_{1}1\\ \vdots \\ r_{n}-{\bar{r}}_{n}1 \end{array}\right]=RC=R\left(I-\frac{E}{n}\right) \end{array}$$

where *diag*(*A*) is a diagonal that shares the same diagonal elements with *A*, and $C=I-\frac{E}{n}$, with *I* and *E* being the identity and all-one matrix, respectively. Therefore, we have $R^{new}=\frac{1}{\sqrt{\left[diag\left(RCR\right)\right]}}\left(RCR\right)\frac{1}{\sqrt{\left[diag\left(RCR\right)\right]}}$, and accordingly have the iterative formula is given as follows below:

(11) $$\begin{array}{r} R_{n}=\frac{1}{\sqrt{\left[diag\left(R_{n-1}CR_{n-1}\right)\right]}}\left(R_{n-1}CR_{n-1}\right)\frac{1}{\sqrt{\left[diag\left(R_{n-1}CR_{n-1}\right)\right]}} \end{array}$$
